# The impact of repeated vaccination using 10-year vaccination history on protection against influenza in older adults: a test-negative design study across the 2010/11 to 2015/16 influenza seasons in Ontario, Canada

**DOI:** 10.2807/1560-7917.ES.2020.25.1.1900245

**Published:** 2020-01-09

**Authors:** Jeffrey C Kwong, Hannah Chung, James KH Jung, Sarah A Buchan, Aaron Campigotto, Michael A Campitelli, Natasha S Crowcroft, Jonathan B Gubbay, Timothy Karnauchow, Kevin Katz, Allison J McGeer, J Dayre McNally, David C Richardson, Susan E Richardson, Laura C Rosella, Kevin L Schwartz, Andrew Simor, Marek Smieja, George Zahariadis, on behalf of the Canadian Immunization Research Network (CIRN) investigators

**Affiliations:** 1 ICES, Toronto, Ontario, Canada; 2 Public Health Ontario, Toronto, Ontario, Canada; 3 Dalla Lana School of Public Health, University of Toronto, Toronto, Ontario, Canada; 4 Department of Family & Community Medicine, University of Toronto, Toronto, Ontario, Canada; 5 Centre for Vaccine Preventable Diseases, University of Toronto, Toronto, Ontario, Canada; 6 University Health Network, Toronto, Ontario, Canada; 7 Hospital for Sick Children, Toronto, Ontario, Canada; 8 London Health Sciences Centre, London, Ontario, Canada; 9 Department of Laboratory Medicine and Pathobiology, University of Toronto, Toronto, Ontario, Canada; 10 Children’s Hospital of Eastern Ontario, Ottawa, Ontario, Canada; 11 Department of Pathology and Laboratory Medicine, University of Ottawa, Ottawa, Ontario, Canada; 12 North York General Hospital, Toronto, Ontario, Canada; 13 Sinai Health System, Toronto, Ontario, Canada; 14 William Osler Health System, Brampton, Ontario, Canada; 15 Sunnybrook Health Sciences Centre, Toronto, Ontario, Canada; 16 McMaster University, Hamilton, Ontario, Canada; 17 Newfoundland & Labrador Public Health Laboratory, St. John’s, Newfoundland and Labrador, Canada; 18 CIRN is acknowledged at the end of the article

**Keywords:** Influenza vaccine, vaccine effectiveness, repeated vaccination, older adults

## Abstract

**Introduction:**

Annual influenza vaccination is recommended for older adults, but evidence regarding the impact of repeated vaccination has been inconclusive.

**Aim:**

We investigated vaccine effectiveness (VE) against laboratory-confirmed influenza and the impact of repeated vaccination over 10 previous seasons on current season VE among older adults.

**Methods:**

We conducted an observational test-negative study in community-dwelling adults aged > 65 years in Ontario, Canada for the 2010/11 to 2015/16 seasons by linking laboratory and health administrative data. We estimated VE using multivariable logistic regression. We assessed the impact of repeated vaccination by stratifying by previous vaccination history.

**Results:**

We included 58,304 testing episodes for respiratory viruses, with 11,496 (20%) testing positive for influenza and 31,004 (53%) vaccinated. Adjusted VE against laboratory-confirmed influenza for the six seasons combined was 21% (95% confidence interval (CI): 18 to 24%). Patients who were vaccinated in the current season, but had received no vaccinations in the previous 10 seasons, had higher current season VE (34%; 95%CI: 9 to 52%) than patients who had received 1–3 (26%; 95%CI: 13 to 37%), 4–6 (24%; 95%CI: 15 to 33%), 7–8 (13%; 95%CI: 2 to 22%), or 9–10 (7%; 95%CI: −4 to 16%) vaccinations (trend test p = 0.001). All estimates were higher after correcting for misclassification of current season vaccination status. For patients who were not vaccinated in the current season, residual protection rose significantly with increasing numbers of vaccinations received previously.

**Conclusions:**

Although VE appeared to decrease with increasing numbers of previous vaccinations, current season vaccination likely provides some protection against influenza regardless of the number of vaccinations received over the previous 10 influenza seasons.

## Introduction

Influenza vaccination is the primary strategy to prevent influenza-related morbidity and mortality, especially for older adults, who are at higher risk of severe outcomes [1]. In this age group, influenza vaccines are 24–63% effective in preventing laboratory-confirmed influenza [2-4]. Due to frequent changes in circulating virus strains, annual vaccination is recommended.

However, the impact of repeated vaccination on vaccine effectiveness (VE) is uncertain. A randomised trial (RCT) conducted in the 1970s at a British boarding school found higher influenza incidence among students who had received multiple previous vaccines than among those who received only the current season’s vaccine [5]. Results from a larger RCT among adults in the 1980s did not lead to the same conclusion [6]. Based on the antigenic distance hypothesis put forth by Smith et al., negative or positive interference can result from prior season vaccination depending on differences in the antigenic distances between prior and current vaccine strains and the current epidemic strain [7]. Most studies to date incorporated only a single previous season when examining the impact of repeated vaccination [8-13]. Meta-analyses of these studies found substantial heterogeneity in repeated vaccination effects [14-16].

Two studies examined the impact of repeated vaccination for five previous seasons. Whereas McLean et al. observed current season VE to be higher in those who were not vaccinated in any of the previous five seasons compared with those who were vaccinated in all five previous seasons [17], Örtqvist et al. found no negative effect of repeated vaccination [18]. Thus, the effect of repeated vaccination beyond one previous season also remains unclear. This is of particular interest for older adults because not only do they bear the greatest burden of disease, but they are also recommended to receive the vaccine annually in most countries and therefore may have received many doses.

The objectives of this study were to estimate VE against laboratory-confirmed influenza infection in community-dwelling older adults for the 2010/11 to 2015/16 seasons and to investigate the impact of repeated vaccination for up to 10 previous seasons on current season VE.

## Methods

### Study population, setting, and design

We studied community-dwelling adults aged > 65 years in Ontario (2016 population aged ≥ 65 years: 2.3 million) who were tested for influenza during inpatient or outpatient healthcare encounters between 1 September 2010 and 31 August 2016. Details regarding these six influenza seasons have been reported previously [19]. We used personal identifiers (health card number, name, date of birth, sex, postal code) and a combination of deterministic and probabilistic methods to link the results of respiratory virus tests performed by a network of 19 public health and academic hospital laboratories to population-based provincial health administrative data (linkage proportion = 97.8%) [19]. These datasets were linked using unique coded identifiers and analysed at ICES (formerly the Institute for Clinical Evaluative Sciences). All patients had universal access to physician services, hospital care, diagnostic testing, prescription medications, and trivalent influenza vaccines during the study.

We estimated VE using the test-negative design, which compares the odds of influenza vaccination among laboratory-confirmed influenza cases and test-negative controls [20].

### Ethical statement

Ethics approval for this study was obtained from the participating laboratories (Supplementary Table S1). The planning, conduct, and reporting of this study was in line with the Declaration of Helsinki.

### Data sources and definitions

#### Laboratory data

We included the results of all respiratory virus tests conducted by participating laboratories. The laboratories used monoplex and multiplex PCR assays, viral culture, direct immunofluorescence assay, or enzyme immunoassay tests to test for one or more of the following viruses: adenovirus, bocavirus, coronavirus, enterovirus/rhinovirus, human metapneumovirus, influenza A, influenza B, parainfluenza virus, and respiratory syncytial virus [19]. We combined the results of all specimens for the same individual on the same day into a single testing episode. For participants tested multiple times in the same season, we included their earliest testing episode positive for influenza (or their earliest testing episode if all specimens tested negative for influenza) for analysis. Individuals tested in multiple seasons contributed one testing episode per season, which were treated as separate units in the analysis. Specimens were submitted at the discretion of clinicians as part of routine clinical care. The proportion of patients presenting with acute respiratory illnesses (ARI) who were tested for influenza varied by setting (22.1% for inpatients, 2.5% for patients in emergency departments, and 2.3% for patients in physician offices) [19].

Since only 49% of individuals positive for influenza A had their specimens subtyped, we assessed their generalisability by comparing the characteristics of those with subtyped and unsubtyped influenza A specimens (Supplementary Table S2). Information on lineage for influenza B was not available.

#### Healthcare encounter data

We identified all healthcare encounters associated with a specimen on the date of collection using the Canadian Institute for Health Information’s Discharge Abstract Database (CIHI-DAD), the National Ambulatory Care Reporting System (NACRS) database, and the Ontario Health Insurance Plan (OHIP) database. The proportion of missing data in each of the healthcare use databases should be very low since healthcare is universally covered for those with provincial health insurance.

#### Influenza vaccination

We ascertained influenza vaccination status using physician and (starting in 2012, when a policy change permitted pharmacists to administer influenza vaccines) pharmacist billing claims, maintained in the OHIP and Ontario Drug Benefit (ODB) databases, respectively. For VE calculations, participants were considered immunised if a vaccine dose was received ≥ 14 days before the specimen collection date.

#### Covariates

We obtained demographic information including age, sex, and census area-level neighbourhood income quintile through the Ontario Registered Persons Database. Healthcare use information including the number of hospitalisations in the past 3 years, outpatient visits in the past year, receipt of home care services in the past year, and prescription medications in the past year were determined using CIHI-DAD, OHIP, Home Care Database, and ODB, respectively. We determined the presence of comorbidities that increase the risk of influenza complications (anaemia, cancer, cardiovascular disease, dementia, diabetes, frailty, immunodeficiency due to underlying disease and/or therapy, as well as renal disease and respiratory disease) based on the presence of these diagnoses in various databases before the date of specimen collection [19].

### Statistical analysis

#### Vaccine effectiveness

We used logistic regression to estimate VE, against laboratory-confirmed influenza infection, by comparing the odds of vaccination in the test-positive cases to the odds of vaccination in the test-negative controls through an odds ratio (OR) and using the following formula VE = (1 − OR) × 100%. The models controlled for the demographic characteristics and measures of previous healthcare use listed above, presence of any comorbidity, calendar time (month of test), and influenza season (except when estimating VE by season). These variables were selected a priori and were included because of their clinical importance and conceptualisation as potential confounders. We used a threshold level of 5% test positivity for the province to restrict the analyses to periods when influenza was circulating.

We estimated VE against any influenza and each influenza type/subtype for the 2010/11 to 2015/16 seasons combined and for each season separately. We also performed subgroup analyses by age group, sex, and healthcare setting, and used interaction tests to assess whether VE differed by subgroup.

We conducted a number of sensitivity analyses. First, we restricted the cohort to patients who had a diagnostic code for an ARI [19] for their healthcare encounter, to emulate case definitions used in prospective test-negative studies. Second, we restricted the cohort to patients who were tested by PCR. Third, since Ontario residents may receive influenza vaccines in settings besides physician offices and pharmacies (leading to incorrectly classifying individuals vaccinated outside of these settings as unvaccinated), for each of the above analyses we conducted a quantitative sensitivity analysis using a publicly available macro [21] to correct for misclassification of influenza vaccination status using previously reported parameters for sensitivity (69%) and specificity (90%) of influenza vaccination codes for older adults in Ontario health administrative databases [22]. This macro performs multiple iterations of exposure re-classification for each execution. For each iteration, sensitivity and specificity values within the 95% confidence interval (CI) of the previously reported parameters are selected. Using these values and the observed counts of exposed cases and controls, expected counts are determined to calculate a positive predictive value (PPV) and negative predictive value (NPV) for cases and controls separately. PPV is the probability that the individual was correctly classified as exposed, whereas NPV is the probability that an individual was correctly classified as unexposed. For each individual, a random number is chosen from a uniform distribution between 0 and 1, and compared with the predictive value based on their case and exposure statuses. If the random number is greater than the predictive value, the subject is reclassified [21]. An OR is calculated using the reclassified exposure value for each iteration, and the median OR from the distribution of ORs from all iterations is reported [21]. This macro does not permit incorporation of interaction terms with the main exposure to do an interaction test between subgroups.

#### Impact of repeated vaccination on current season vaccine effectiveness

Next, we examined the impact of repeated vaccination on current season VE against any influenza and each influenza type/subtype for the 2010/11 to 2015/16 seasons combined. We did this taking into account incrementally longer vaccination history durations (i.e. one, five, and 10 previous influenza seasons). Since influenza vaccination data in Ontario are more accurate among those aged ≥ 65 years [22], we restricted the analysis examining 5-year vaccination history to patients aged ≥ 70 years in the current season to ensure they were ≥ 65 years for all previous seasons. Similarly, we restricted the analysis examining 10-year vaccination history to patients aged ≥ 75 years in the current season. Patients had to be eligible for health insurance in Ontario during the previous seasons investigated.

For each analysis, we stratified the study population based on individuals’ vaccination history (i.e. number of previous vaccinations received) and we estimated current season VE conditioned on vaccination history. Therefore, the reference group for estimating VE is patients who share similar vaccination histories as those who are vaccinated in the current season but are unvaccinated in the current season. For example, we compared patients who had received 9–10 previous vaccinations and who were vaccinated in the current season to those who had received 9–10 previous vaccinations but who were not vaccinated in the current season. The rationale for this approach is that it quantifies the incremental benefit of vaccination in the current season, and acknowledges that since a patient cannot change his/her past vaccination status, comparing to those not vaccinated in the current nor any past season may not be appropriate. Ultimately, this provides more patient-centred results as it aligns with the decision that needs to be made by patients each season regarding the benefit of receiving the current season’s vaccine.

We used interaction tests to assess differences in current season VE estimates between those vaccinated in the prior season and those not vaccinated in the prior season. For previous vaccination histories of five and 10 seasons, we used meta-regression to assess for trends in VE estimates between the vaccination history strata [23].

In sensitivity analyses, we corrected for misclassification of current season vaccination status. For the macro programme to successfully execute, we assumed the same values of sensitivity and specificity for all strata of past vaccination history. We repeated the analyses restricted to patients aged ≥ 75 years in the current season for greater consistency of the VE estimates across the varying vaccination history durations. We also conducted sensitivity analyses in which we manually reclassified past vaccination status for those who were misclassified for the current season based on the macro programme (details in Supplementary Text). In the first scenario, we changed vaccination status from unvaccinated to vaccinated for all previous seasons, effectively moving all misclassified individuals into the most vaccinated category in terms of vaccination history. In the second scenario, we moved individuals ‘up’ a single category (e.g. for the analysis examining 5-year vaccination history, those initially considered vaccinated in none of the previous five seasons were re-categorised to the ‘vaccinated in 1–3 of the previous five seasons’ group).

To facilitate comparisons with previous studies, we repeated these analyses using the conventional approach of estimating VE for all combinations of vaccine exposure in the current and previous seasons against a common reference group of patients who were not vaccinated in the current season and any previous seasons under consideration. To assess for trend with this approach, we included the parameterised vaccination history variable as a continuous variable in the model [24].

#### Analysis tools and statistical significance

Analyses were conducted using SAS version 9.4 (SAS Institute, Cary, NC) and R version 3.4.0 (R Core Team, Vienna, Austria). All tests were two-sided and used p *<* 0.05 as the level of statistical significance.

## Results

We included 58,304 testing episodes (obtained from 54,116 unique patients, including 7% tested during multiple seasons), with 11,496 (20%) testing positive for influenza and 31,004 (53%) vaccinated during the season of testing and before specimen collection. Compared with test-negative controls, test-positive cases were older, were more likely to be female, used fewer health services, had fewer comorbidities, and were less likely to be vaccinated (Supplementary Table S3). Descriptive statistics comparing vaccinated and unvaccinated patients can be found in Supplementary Table S4.

Overall adjusted VE against any influenza for the 2010/11 to 2015/16 seasons combined was 21% (95%CI: 18 to 24%) (**Table 1**). For the six seasons combined, VE was 38% (95%CI: 28 to 46%) against A(H1N1)pdm09, 22% (95%CI: 16 to 28%) against A(H3N2), and 30% (95%CI: 24 to 36%) against B. VE against unsubtyped influenza A viruses was only 11% (95%CI: 5 to 16%). We observed substantial variability in VE by season (interaction test p < 0.001), by age group (p = 0.01), and by sex (p = 0.03), but not by healthcare setting (p = 0.60). After correcting for misclassification of vaccination status, VE for the six seasons combined increased to 38% (95%CI: 35 to 42%) against any influenza. We observed similar results when restricting the analysis to ARI-coded healthcare encounters and to patients tested by PCR (**Table 1**). VE estimates stratified by influenza subtype and season are presented in Supplementary Table S5.

**Table 1:** Influenza vaccine effectiveness estimates for community-dwelling adults aged > 65 years, 2010/11 to 2015/16 influenza seasons in Ontario, Canada (n = 58,304)^a^

Analysis		Test-positive patients No. vaccinated/total	Test-negative patients No. vaccinated/total	Unadjusted VE% (95% CI)	Adjusted VE% (95% CI)	Misclassification corrected Adjusted VE% (95% CI)
Overall^b^		5,575/11,496	25,429/46,808	21 (18 to 24)	21 (18 to 24)	38 (35 to 42)
By influenza type/subtype						
Influenza A^c^	A(H3N2)	1,780/3,765	25,429/46,808	25 (19 to 29)	22 (16 to 28)	44 (38 to 49)
	A(H1N1)pdm09	347/830	25,429/46,808	40 (31 to 47)	38 (28 to 46)	61 (53 to 68)
	A(unsubtyped)	2,425/4,772	25,429/46,808	13 (8 to 18)	11 (5 to 16)	25 (18 to 31)
Influenza B		1,027/2,138	25,429/46,808	22 (15 to 29)	30 (24 to 36)	42 (37 to 49)
By influenza season						
2010/11		488/1,204	2,561/4,980	36 (27 to 43)	33 (23 to 41)	54 (45 to 61)
2011/12		195/413	1,823/3,216	32 (16 to 44)	32 (16 to 45)	54 (34 to 66)
2012/13		988/2,253	4,339/8,577	24 (16 to 31)	20 (12 to 28)	38 (29 to 45)
2013/14		711/1,554	5,368/9,665	32 (25 to 39)	36 (28 to 42)	56 (49 to 62)
2014/15		2,416/4,432	6,712/12,044	5 (−2 to 11)	6 (−1 to 13)	12 (2 to 21)
2015/16		777/1,640	4,626/8,326	28 (20 to 35)	26 (17 to 34)	49 (40 to 56)
By age group in years						
66–75		1,525/3,601	8,387/16,716	27 (22 to 32)	28 (22 to 33)	42 (34 to 49)
76–85		2,328/4,548	10,402/18,181	22 (16 to 27)	20 (14 to 25)	42 (36 to 48)
≥ 86		1,722/3,347	6,640/11,911	16 (9 to 22)	13 (5 to 20)	31 (22 to 38)
By sex						
Male		2,611/5,348	12,470/22,446	24 (19 to 28)	25 (21 to 30)	44 (38 to 49)
Female		2,964/6,148	12,959/24,362	18 (13 to 23)	17 (12 to 22)	34 (28 to 40)
By healthcare setting						
Inpatient		4,460/9,224	22,424/41,178	22 (18 to 25)	21 (18 to 25)	40 (35 to 44)
Outpatient		1,146/2,330	3,398/6,368	15 (7 to 23)	18 (10 to 26)	30 (20 to 39)
ARI-coded encounter		5,068/10,459	16,968/31,185	21 (18 to 25)	21 (18 to 25)	39 (35 to 43)
Tested by PCR^d^		4,741/9,841	19,586/35,877	23 (19 to 26)	22 (18 to 26)	41 (37 to 45)

ARI: acute respiratory illness; CI: confidence interval; No.: number; NA: not applicable; VE: vaccine effectiveness.

**^a^** The model adjusted for age, sex, census area-level neighbourhood income quintile, number of hospitalisations in the past 3 years, number of outpatient visits in the past year, receipt of home care services in the past year, number of prescription medications in the past year, comorbidities that increase the risk of influenza complications (anaemia, cancer, cardiovascular disease, dementia, diabetes, frailty, immunodeficiency due to underlying disease and/or therapy, as well as renal disease and respiratory disease), calendar time, and influenza season.

**^b^** For any influenza, 2010/11 to 2015/16 influenza seasons combined.

**^c^** Only 49% of influenza A specimens were subtyped.

**^d^** PCR (monoplex or multiplex).

### Impact of repeated vaccination on vaccine effectiveness

**Figure 1:**
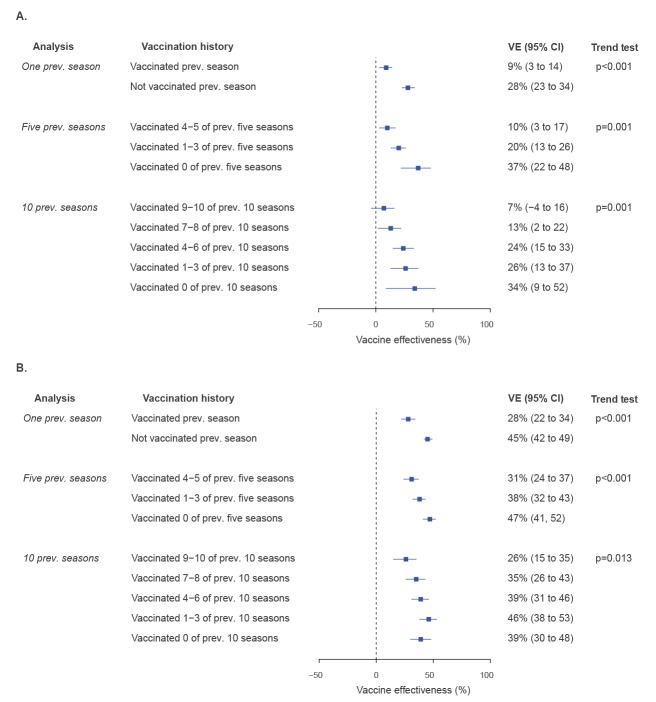
Forest plots of (A) current season vaccine effectiveness estimates against any influenza for community-dwelling adults aged > 65 years, taking into account vaccination histories for one, five, and 10 previous seasons and stratifying according to number of vaccinations received and (B) also correcting for misclassification of current season vaccination status, Ontario, Canada

Patients who had received more vaccinations in previous seasons were older and more likely to be male, use health services, and have comorbidities, although the magnitudes of the differences between groups were small (**Table 2**, **Table 3**, **Table 4**). Current season adjusted VE was higher for patients not vaccinated in the previous season (28%; 95%CI: 23 to 34%) than for those who were vaccinated in the previous season (9%; 95%CI: 3 to 14%) (interaction test p < 0.001) (**Figure 1a**). In the analysis accounting for 5-year vaccination history, patients who had received no vaccinations in the previous five seasons had the highest VE for current season vaccination (37%; 95%CI: 22 to 48%), with lower but still significant VE estimates for patients who had received 1–3 (20%; 95%CI: 13 to 26%) and 4–5 (10%; 95%CI: 3 to 17%) vaccinations in the previous five seasons (trend test p = 0.001). Similar results were observed when accounting for 10-year vaccination history: patients who had received no vaccinations in the previous 10 seasons had the highest VE for current season vaccination (34%; 95%CI: 9 to 52%), with VE decreasing with more previous vaccinations received over the previous 10 seasons: 26% (95%CI: 13 to 37%) for those vaccinated 1–3 times, 24% (95%CI: 15 to 33%) for those vaccinated 4–6 times, 13% (95%CI: 2 to 22%) for those vaccinated 7–8 times, and 7% (95%CI: −4 to 16%) for those vaccinated 9–10 times (trend test p = 0.001).

**Table 2:** Descriptive characteristics of community-dwelling adults aged > 65 years for the 2010/11 to 2015/16 influenza seasons, stratified by vaccination history for the previous season, Ontario, Canada (n = 58,021)

Characteristic	Total (n = 58,021)		Vaccinated in previous season (n = 33,243)		Not vaccinated in previous season (n = 24,778)		p value
	Number	%	Number	%	Number	%	
Influenza season							
2010/11	6,162	10.6	3,678	11.1	2,484	10.0	< 0.001
2011/12	3,607	6.2	2,081	6.3	1,526	6.2	
2012/13	10,777	18.6	5,874	17.7	4,903	19.8	
2013/14	11,145	19.2	6,079	18.3	5,066	20.4	
2014/15	16,411	28.3	9,743	29.3	6,668	26.9	
2015/16	9,919	17.1	5,788	17.4	4,131	16.7	
Age (years), mean ± SD	79.6 ± 8.2	NA	80.1 ± 8.0	NA	78.8 ± 8.4	NA	< 0.001
Age group in years							
66–75	20,192	34.8	10,495	31.6	9,697	39.1	< 0.001
76–85	22,617	39.0	13,588	40.9	9,029	36.4	
≥ 86	15,212	26.2	9,160	27.6	6,052	24.4	
Male sex	27,660	47.7	16,139	48.5	11,521	46.5	< 0.001
Neighbourhood income quintile							
1 (lowest)	13,044	22.5	7,113	21.4	5,931	23.9	< 0.001
2	12,321	21.2	7,112	21.4	5,209	21.0	
3	10,935	18.8	6,300	19.0	4,635	18.7	
4	10,341	17.8	6,001	18.1	4,340	17.5	
5 (highest)	11,026	19.0	6,540	19.7	4,486	18.1	
Missing	354	0.6	177	0.5	177	0.7	
Medical conditions							
Cardiovascular disease^a^	37,212	64.1	21,778	65.5	15,434	62.3	< 0.001
Chronic obstructive pulmonary disease	29,672	51.1	17,734	53.3	11,938	48.2	< 0.001
Diabetes	24,858	42.8	14,594	43.9	10,264	41.4	< 0.001
Cancer	17,082	29.4	10,167	30.6	6,915	27.9	< 0.001
Asthma	16,179	27.9	9,948	29.9	6,231	25.1	< 0.001
Anaemia	13,988	24.1	8,384	25.2	5,604	22.6	< 0.001
Chronic kidney disease	12,853	22.2	7,456	22.4	5,397	21.8	0.063
Dementia/frailty	11,410	19.7	6,481	19.5	4,929	19.9	0.23
Immunocompromised	8,185	14.1	4,983	15.0	3,202	12.9	< 0.001
Any of the above medical conditions	55,351	95.4	32,009	96.3	23,342	94.2	< 0.001
Received homecare services, past 1y	28,321	48.8	16,098	48.4	12,223	49.3	0.03
Hospitalisations, past 3y, mean ± SD	1.6 ± 2.2	NA	1.6 ± 2.1	NA	1.6 ± 2.3	NA	< 0.001
Outpatient visits, past 1y, mean ± SD	14.2 ± 11.0	NA	15.5 ± 11.0	NA	12.5 ± 10.7	NA	< 0.001
Prescription medications, past 1y, mean ± SD	16.5 ± 9.3	NA	17.4 ± 9.1	NA	15.4 ± 9.5	NA	< 0.001
Month of influenza testing							
November	1,407	2.4	785	2.4	622	2.5	0.83
December	9,486	16.3	5,402	16.3	4,084	16.5	
January	15,038	25.9	8,605	25.9	6,433	26.0	
February	10,304	17.8	5,904	17.8	4,400	17.8	
March	10,686	18.4	6,166	18.5	4,520	18.2	
April	7,599	13.1	4,378	13.2	3,221	13.0	
May	3,501	6.0	2,003	6.0	1,498	6.0	
Tested sample from inpatient setting	49,621	85.5	28,544	85.9	21,077	85.1	0.007
Specimen positive for influenza	11,444	19.7	6,177	18.6	5,267	21.3	< 0.001
Vaccinated against influenza in current season	30,916	53.3	24,592	74.0	6,324	25.5	< 0.001

NA: not applicable; SD: standard deviation.

**^a^** Includes acute ischaemic stroke, arrhythmias, congestive heart failure, ischaemic heart disease, and transient ischaemic attack.

**Table 3:** Descriptive characteristics of community-dwelling adults aged ≥ 70 years for the 2010/11 to 2015/16 influenza seasons, stratified by influenza vaccination history over five previous seasons, Ontario, Canada (n = 49,294)

Characteristic	Vaccination history over five previous seasons								
	Total (n = 49,294)		4–5 vaccinations (n = 24,664)		1–3 vaccinations (n = 15,933)		0 vaccinations (n = 8,697)		p value
	Number	%	Number	%	Number	%	Number	%	
Influenza season									
2010/11	5,295	10.7	2,656	10.8	1,700	10.7	939	10.8	0.05
2011/12	3,046	6.2	1,589	6.4	932	5.8	525	6.0	
2012/13	9,216	18.7	4,563	18.5	3,023	19.0	1,630	18.7	
2013/14	9,328	18.9	4,609	18.7	3,011	18.9	1,708	19.6	
2014/15	14,192	28.8	7,178	29.1	4,618	29.0	2,396	27.5	
2015/16	8,217	16.7	4,069	16.5	2,649	16.6	1,499	17.2	
Age (years), mean ± SD	81.5 ± 7.1	NA	82.1 ± 6.9	NA	81.3 ± 7.2	NA	80.3 ± 7.1	NA	< 0.001
Age group in years									
70–75	11,952	24.2	5,058	20.5	4,191	26.3	2,703	31.1	< 0.001
76–85	22,290	45.2	11,553	46.8	6,963	43.7	3,774	43.4	
≥ 86	15,052	30.5	8,053	32.7	4,779	30.0	2,220	25.5	
Male sex	23,256	47.2	11,936	48.4	7,386	46.4	3,934	45.2	< 0.001
Neighbourhood income quintile									
1 (lowest)	10,872	22.1	5,102	20.7	3,652	22.9	2,118	24.4	< 0.001
2	10,485	21.3	5,349	21.7	3,316	20.8	1,820	20.9	
3	9,356	19.0	4,690	19.0	3,068	19.3	1,598	18.4	
4	8,829	17.9	4,458	18.1	2,824	17.7	1,547	17.8	
5 (highest)	9,470	19.2	4,934	20.0	2,994	18.8	1,542	17.7	
Missing	282	0.6	131	0.5	79	0.5	72	0.8	
Medical conditions									
Cardiovascular disease^a^	32,830	66.6	16,841	68.3	10,610	66.6	5,379	61.8	< 0.001
Chronic obstructive pulmonary disease	25,351	51.4	13,214	53.6	8,329	52.3	3,808	43.8	< 0.001
Diabetes	21,154	42.9	10,910	44.2	6,850	43.0	3,394	39.0	< 0.001
Cancer	14,559	29.5	7,563	30.7	4,682	29.4	2,314	26.6	< 0.001
Asthma	13,725	27.8	7,561	30.7	4,381	27.5	1,783	20.5	< 0.001
Anaemia	12,090	24.5	6,375	25.8	3,899	24.5	1,816	20.9	< 0.001
Chronic kidney disease	11,272	22.9	5,706	23.1	3,804	23.9	1,762	20.3	< 0.001
Dementia/frailty	10,845	22.0	5,345	21.7	3,877	24.3	1,623	18.7	< 0.001
Immunocompromised	6,463	13.1	3,433	13.9	2,060	12.9	970	11.2	< 0.001
Any of the above medical conditions	47,310	96.0	23,877	96.8	15,335	96.2	8,098	93.1	< 0.001
Received homecare services, past 1y	25,184	51.1	12,567	51.0	8,456	53.1	4,161	47.8	< 0.001
Hospitalisations, past 3y, mean ± SD	1.6 ± 2.1	NA	1.5 ± 2.1	NA	1.7 ± 2.3	NA	1.4 ± 2.1	NA	< 0.001
Outpatient visits, past 1y, mean ± SD	14.0 ± 10.7	NA	15.5 ± 10.7	NA	13.3 ± 10.6	NA	11.0 ± 9.9	NA	< 0.001
Prescription medications, past 1y, mean ± SD	16.6 ± 9.1	NA	17.5 ± 8.8	NA	16.8 ± 9.2	NA	13.7 ± 9.0	NA	< 0.001
Month of influenza testing									
November	1,178	2.4	590	2.4	380	2.4	208	2.4	0.74
December	8,127	16.5	4,111	16.7	2,592	16.3	1,424	16.4	
January	12,920	26.2	6,376	25.9	4,250	26.7	2,294	26.4	
February	8,741	17.7	4,346	17.6	2,813	17.7	1,582	18.2	
March	9,009	18.3	4,533	18.4	2,939	18.4	1,537	17.7	
April	6,398	13.0	3,237	13.1	2,024	12.7	1,137	13.1	
May	2,921	5.9	1,471	6.0	935	5.9	515	5.9	
Tested sample from inpatient setting	42,652	86.5	21,487	87.1	13,767	86.4	7,398	85.1	< 0.001
Specimen positive for influenza	9,877	20.0	4,743	19.2	3,117	19.6	2,017	23.2	< 0.001
Vaccinated against influenza in current season	27,043	54.9	18,946	76.8	7,325	46.0	772	8.9	< 0.001

NA: not applicable; SD: standard deviation.

**^a^** Includes acute ischaemic stroke, arrhythmias, congestive heart failure, ischaemic heart disease, and transient ischaemic attack.

**Table 4:** Descriptive characteristics of community-dwelling adults aged ≥ 75 years for the 2010/11 to 2015/16 influenza seasons, stratified by influenza vaccination history over 10 previous seasons, Ontario, Canada (n = 38,766)

Characteristic	Vaccination history over 10 previous seasons												
	Total (n = 38,766)		9–10 vaccinations (n = 13,036)		7–8 vaccinations (n = 9,008)		4–6 vaccinations (n = 7,416)		1–3 vaccinations (n = 5,147)		0 vaccinations (n = 4,159)		p value
	Number	%	Number	%	Number	%	Number	%	Number	%	Number	%	
Influenza season													
2010/11	4,144	10.7	1,268	9.7	1,024	11.4	852	11.5	539	10.5	461	11.1	0.02
2011/12	2,371	6.1	835	6.4	552	6.1	442	6.0	297	5.8	245	5.9	
2012/13	7,294	18.8	2,430	18.6	1,702	18.9	1,407	19.0	948	18.4	807	19.4	
2013/14	7,254	18.7	2,470	18.9	1,663	18.5	1,363	18.4	974	18.9	784	18.9	
2014/15	11,416	29.4	3,953	30.3	2,595	28.8	2,133	28.8	1,552	30.2	1,183	28.4	
2015/16	6,287	16.2	2,080	16.0	1,472	16.3	1,219	16.4	837	16.3	679	16.3	
Age (years), mean ± SD	83.9 ± 5.8	NA	84.4 ± 5.7	NA	84.3 ± 5.8	NA	83.6 ± 5.8	NA	83.2 ± 5.8	NA	83.1 ± 5.9	NA	< 0.001
Age group in years													
75	2,011	5.2	509	3.9	410	4.6	442	6.0	343	6.7	307	7.4	< 0.001
76–85	21,900	56.5	7,149	54.8	4,943	54.9	4,273	57.6	3,056	59.4	2,479	59.6	
≥ 86	14,855	38.3	5,378	41.3	3,655	40.6	2,701	36.4	1,748	34.0	1,373	33.0	
Male sex	17,936	46.3	6,291	48.3	4,060	45.1	3,408	46.0	2,358	45.8	1,819	43.7	< 0.001
Neighbourhood income quintile													
1 (lowest)	8,338	21.5	2,592	19.9	1,883	20.9	1,664	22.4	1,194	23.2	1,005	24.2	< 0.001
2	8,264	21.3	2,846	21.8	1,876	20.8	1,588	21.4	1,090	21.2	864	20.8	
3	7,328	18.9	2,509	19.2	1,695	18.8	1,410	19.0	1,007	19.6	707	17.0	
4	6,988	18.0	2,350	18.0	1,671	18.6	1,321	17.8	889	17.3	757	18.2	
5 (highest)	7,618	19.7	2,661	20.4	1,841	20.4	1,396	18.8	942	18.3	778	18.7	
Missing	230	0.6	78	0.6	42	0.5	37	0.5	25	0.5	48	1.2	
Medical conditions													
Cardiovascular disease^a^	26,959	69.5	9,175	70.4	6,453	71.6	5,230	70.5	3,415	66.3	2,686	64.6	< 0.001
Chronic obstructive pulmonary disease	19,818	51.1	6,819	52.3	4,809	53.4	3,935	53.1	2,537	49.3	1,718	41.3	< 0.001
Diabetes	16,241	41.9	5,634	43.2	3,889	43.2	3,115	42.0	2,096	40.7	1,507	36.2	< 0.001
Cancer	11,339	29.2	4,011	30.8	2,655	29.5	2,185	29.5	1,409	27.4	1,079	25.9	< 0.001
Asthma	10,623	27.4	3,896	29.9	2,671	29.7	2,042	27.5	1,229	23.9	785	18.9	< 0.001
Anaemia	9,559	24.7	3,428	26.3	2,309	25.6	1,764	23.8	1,181	22.9	877	21.1	< 0.001
Chronic kidney disease	9,040	23.3	2,997	23.0	2,241	24.9	1,833	24.7	1,145	22.2	824	19.8	< 0.001
Dementia/frailty	9,795	25.3	3,129	24.0	2,494	27.7	2,022	27.3	1,298	25.2	852	20.5	< 0.001
Immunocompromised	4,514	11.6	1,551	11.9	1,124	12.5	937	12.6	529	10.3	373	9.0	< 0.001
Any of the above medical conditions	37,380	96.4	12,645	97.0	8,743	97.1	7,182	96.8	4,927	95.7	3,883	93.4	< 0.001
Received homecare services, past 1y	21,006	54.2	6,947	53.3	5,087	56.5	4,148	55.9	2,734	53.1	2,090	50.3	< 0.001
Hospitalisations, past 3y, mean ± SD	1.5 ± 2.1	NA	1.5 ± 1.9	NA	1.7 ± 2.2	NA	1.7 ± 2.2	NA	1.6 ± 2.1	NA	1.3 ± 1.8	NA	< 0.001
Outpatient visits, past 1y, mean ± SD	13.7 ± 10.3	NA	15.3 ± 10.3	NA	14.2 ± 10.4	NA	13.1 ± 10.3	NA	12.0 ± 10.0	NA	10.5 ± 9.3	NA	< 0.001
Prescription medications, past 1y, mean ± SD	16.5 ± 8.8	NA	17.2 ± 8.5	NA	17.4 ± 8.8	NA	16.8 ± 9.0	NA	15.4 ± 8.8	NA	13.1 ± 8.8	NA	< 0.001
Month of influenza testing													
November	908	2.3	286	2.2	232	2.6	161	2.2	146	2.8	83	2.0	0.27
December	6,426	16.6	2,226	17.1	1,479	16.4	1,217	16.4	834	16.2	670	16.1	
January	10,301	26.6	3,412	26.2	2,352	26.1	2,041	27.5	1,364	26.5	1,132	27.2	
February	6,853	17.7	2,263	17.4	1,620	18.0	1,300	17.5	924	18.0	746	17.9	
March	7,036	18.1	2,352	18.0	1,672	18.6	1,346	18.1	930	18.1	736	17.7	
April	4,976	12.8	1,694	13.0	1,152	12.8	930	12.5	654	12.7	546	13.1	
May	2,266	5.8	803	6.2	501	5.6	421	5.7	295	5.7	246	5.9	
Tested sample from inpatient setting	33,904	87.5	11,474	88.0	7,875	87.4	6,511	87.8	4,449	86.4	3,595	86.4	0.01
Specimen positive for influenza	8,043	20.7	2,653	20.4	1,783	19.8	1,502	20.3	1,095	21.3	1,010	24.3	< 0.001
Vaccinated against influenza in current season	21,645	55.8	10,297	79.0	5,847	64.9	3,768	50.8	1,450	28.2	283	6.8	< 0.001

NA: not applicable; SD: standard deviation.

**^a^** Includes acute ischaemic stroke, arrhythmias, congestive heart failure, ischemic heart disease, and transient ischaemic attack.

CI: confidence interval; prev.: previous; VE: vaccine effectiveness.

^a^ The model adjusted for age, sex, census area-level neighbourhood income quintile, number of hospitalisations in the past 3 years, number of outpatient visits in the past year, receipt of home care services in the past year, number of prescription medications in the past year, comorbidities that increase the risk of influenza complications (anaemia, cancer, cardiovascular disease, dementia, diabetes, frailty, immunodeficiency due to underlying disease and/or therapy, as well as renal disease and respiratory disease), calendar time, and influenza season.

We observed similar trends against A(H3N2) (Supplementary Figure S1) but not against A(H1N1)pdm09 (Supplementary Figure S2) or influenza B (Supplementary Figure S3).

When correcting for misclassification of current season vaccination status, we found similar patterns as the primary analysis, but with VE estimates that were higher in magnitude (**Figure 1b**). Repeating the analyses restricted to patients aged ≥ 75 years in the current season, the patterns were similar to our primary analysis, but the VE estimates were slightly lower (Supplementary Figure S4a). After correcting for misclassification of current season vaccination status within this restricted cohort, VE estimates were higher but the overall trends were consistent (Supplementary Figure S4b). Results were similar when manually reclassifying vaccination status in past seasons based on current season misclassification (Supplementary Figure S5).

Using the conventional approach of comparing to a common reference group, VE did not differ substantially for patients vaccinated in both prior and current seasons (25%; 95%CI: 22 to 29%) and those vaccinated in the current season only (29%; 95%CI: 23 to 34%) (interaction test p = 0.31), but was lower for those vaccinated in the prior season only (18%; 95%CI: 13 to 23%) (p < 0.001) (Supplementary Figure S6). When accounting for 5-year vaccination history, significant protection against influenza was observed among patients with any previous vaccination, with or without current season vaccination. Notably, for similar levels of vaccination in previous seasons, receipt of current season vaccination was associated with higher VE estimates than being unvaccinated in the current season. VE decreased for current vaccine recipients as the number of previous vaccinations received increased (i.e. 36% vs 31% vs 26%) (trend test p = 0.007). In contrast, for those not vaccinated in the current season, residual protection increased as the number of previous vaccinations received increased (i.e. 13% vs 17%) (p < 0.001). Similar patterns were observed when considering 10-year vaccination history, with VE decreasing for current vaccine recipients with increasing numbers of previous vaccinations (from 33% to 22%) (p < 0.001), while the opposite trend in residual protection was observed for those without current season vaccination (from 9% to 16%) (p < 0.001).

## Discussion

In this study of older adults, we estimated VE against laboratory-confirmed influenza healthcare use to be 21% (95%CI: 18 to 24%) during the 2010/11 to 2015/16 influenza seasons, which increased to 38% (95%CI: 35 to 42%) after correcting for misclassification of vaccination status. When we examined the impact of repeated vaccination during previous influenza seasons on VE for the current season, we observed a declining trend in VE as the number of previous vaccinations increased. Nevertheless, influenza vaccination during the current season was associated with some protection against influenza infection irrespective of the number of vaccinations over the previous 10 seasons, except for individuals vaccinated 9–10 times before we corrected for misclassification of vaccination status. After correcting for misclassification of vaccination status in the current season, influenza vaccination was associated with some protection even for those vaccinated 9–10 times during the previous 10 seasons. Reassuringly, the overall observed trends in VE were consistent when correcting for this misclassification, both for the current season only and when manually reclassifying vaccination status during past seasons based on current season misclassification. Similar patterns as any influenza were observed against A(H3N2) but not A(H1N1)pdm09 or influenza B, but interpretation of these results is challenging due to lower case counts for the latter analyses leading to less precision. The observed patterns for any influenza were likely driven by A(H3N2) since that subtype comprised 67% of specimens during the influenza seasons included in this study, if one assumes the subtype distribution for unsubtyped specimens is the same as for subtyped specimens. We noted that for patients who were not vaccinated in the current season, residual protection appeared to increase with increasing numbers of vaccines received during previous seasons. We also demonstrated that being vaccinated in the current season resulted in consistently greater protection, compared with not being vaccinated in the current season, regardless of the number of previous vaccinations.

While reduced VE from repeated vaccination has been reported previously [13,25], this has not been consistently found [11,12]. The antigenic distance hypothesis is one potential explanation for reduced VE; if the vaccine strains for the current season and prior season are similar but the current season’s vaccine strain is distinct from the current epidemic strain, negative interference leading to reduced VE for the current season may result [7]. However, this hypothesis does not consider the effects of multiple previous vaccine or virus exposures [25]. Thompson et al. [26] examined up to 4 years of previous vaccination history among healthcare workers and observed a greater blunting of serologic response to the A(H3N2) vaccine strain with more doses of previous vaccines received. In addition, two studies have examined vaccination history for up to five previous seasons, with one study observing reduced VE with repeated vaccination [17] while the other did not [18]. However, similar to our results, both studies showed that vaccination in the current season provided some protection against influenza regardless of the number of previous vaccinations. No study has ever examined the impact of repeated vaccination over 10 previous seasons. The residual protection from being vaccinated in previous seasons observed in our study has also been seen elsewhere; this phenomenon may result from cross-reactivity of immune responses elicited by previous vaccinations with current-season virus antigens [17]. It is possible that due to this potential residual protection, the incremental benefit of current season vaccination may be difficult to observe for those who have received many previous vaccinations.

It remains unclear whether true vaccine interference is occurring from repeated vaccination or whether the differences between studies are an artefact of residual confounding [17]. Individuals may be more inclined to be vaccinated for the first time if they were infected by influenza in the prior season. Vaccine responses may be enhanced with recent prior infection [27], such that those who were vaccinated repeatedly may appear to have lower VE. However, measuring immunity arising from previous infection is challenging [11]. In addition, while pooling of multiple seasons can increase statistical power, it can mask important variation at the individual season level [14]. Thus, a large knowledge gap persists regarding the immunologic mechanisms for potential vaccine interference. Future studies that longitudinally ascertain both influenza vaccination and influenza infection status over multiple seasons would be helpful to better understand the impact of repeated vaccination on current season VE as well as residual protection from previous vaccination.

This study has several limitations. First, the specimens were not collected through systematic screening and enrollment but rather as part of routine clinical care. However, we have validated the use of these specimens for estimating VE [19]. Second, while test-negative studies typically use symptom onset date as the index date, we were limited to using specimen collection date. This may have led to underestimation of VE. Third, the VE estimate against unsubtyped influenza A was outside of the range between those against A(H1N1)pdm09 and A(H3N2), raising the possibility of potential bias. Although only 49% of individuals positive for influenza A had their specimens subtyped, they are fairly representative of all individuals positive for influenza A (Supplementary Table S1). We speculate that the lower VE observed for unsubtyped specimens may be due to a greater proportion being collected during the 2014/15 season (a season with known poor match) and during later months of influenza season (with potentially lower VE due to intra-season waning of immunity). Fourth, receipt of influenza vaccines outside of physician offices and pharmacies leads to misclassification of vaccination status when relying on health administrative data to ascertain vaccination status. However, healthcare-seeking behaviour has been found to be similar between test-positive and test-negative individuals [28], so any misclassification would likely be non-differential and underestimate VE, as demonstrated in our sensitivity analyses. The low sensitivity value for the influenza vaccination billing claims used in these sensitivity analyses may have resulted from bias given the self-reported nature of the reference standard available for validation of the claims data, or because pharmacist billing claims data were not yet available for inclusion in the validation study [22]. Fifth, the macro programme used for our sensitivity analysis could not include interaction terms with the main exposure to determine whether subgroup similarities/differences in VE were maintained after misclassification was corrected, and could only correct for misclassification of current season vaccination status and not misclassification in past seasons. However, our sensitivity analyses involving reclassification of past vaccination based on misclassification of current season vaccination status found very similar trends as when accounting only for current season misclassification. Sixth, we used the same values of sensitivity and specificity of the vaccination billing claims for all strata of past vaccination history because we did not have stratum-specific parameters. The observed trend might not remain if the magnitude of the bias correction varies by past vaccination history. Seventh, our use of meta-regression to assess for trends in VE estimates between vaccination history strata does not capture season-to-season heterogeneity in terms of circulating viruses, vaccine match, and host-virus immunological interactions. Eighth, we did not have information on participants’ influenza infections in the previous seasons, as most infections do not result in laboratory testing. Ninth, we did not have information on vaccination history for > 10 previous influenza seasons; the impact of repeated vaccination over longer periods of time remain unknown. Finally, as an observational study, the possibility of residual confounding remains.

## Conclusions

In summary, we observed modest VE for community-dwelling older adults in Ontario, Canada during the 2010/11 to 2015/16 influenza seasons. We observed declining VE associated with repeated vaccination, however current season vaccination likely provides some protection against influenza regardless of the number of vaccines received over the previous 10 influenza seasons. Moreover, among those not vaccinated in the current season, increasing residual protection was observed with increasing numbers of previous vaccines received. Therefore, until effective universal influenza vaccines are available and eliminate the need for annual influenza vaccination, our findings support current recommendations for annual vaccination among older adults given their higher risk for influenza-related morbidity and mortality.

## Supplementary Data

2090000 Supplementary Material
