# Supplementary material for: The impact of repeated vaccination using 10-year vaccination history on protection against influenza in older adults: a test-negative design study across the 2010/11 to 2015/16 influenza seasons in Ontario, Canada
Source: Euro Surveill. 2020 Jan 9;25(1):1900245. doi: 10.2807/1560-7917.ES.2020.25.1.1900245 (PMC6961264; doi:10.2807/1560-7917.ES.2020.25.1.1900245)
Supplement: Supplementary Material [file 1900245_KWONG_Supplementary_Material.pdf]

Supplementary Appendix

This supplementary material is hosted by Eurosurveillance as supporting information alongside the article, “The impact of repeated vaccination using 10-year vaccination history on protection against influenza in older adults: a test-negative design study across the 2010/11 to 2015/16 influenza seasons in Ontario, Canada,” on behalf of the authors who remain responsible for the accuracy and appropriateness of the content. The same standards for ethics, copyright, attributions and permissions as for the article apply. Supplements are not edited by Eurosurveillance and Eurosurveillance is not responsible for the maintenance of any links or email addresses provided therein.

Table of Contents

3. Table S1. Participating laboratories and research ethics approval numbers

4. Table S2. Descriptive characteristics of subtyped and unsubtyped influenza A specimens obtained from community-dwelling adults aged > 65 years, 2010/11 to 2015/16 influenza seasons

5. Supplementary Text. Sensitivity analysis involving manual reclassification of past vaccination status based on misclassification of current season vaccination status

6. Table S3. Descriptive characteristics of influenza test-positive and influenza test-negative community-dwelling adults aged > 65 years, 2010/11 to 2015/16 influenza seasons

7. Table S4. Descriptive characteristics of vaccinated and unvaccinated community-dwelling adults aged > 65 years, 2010/11 to 2015/16 influenza seasons

8. Table S5. Influenza vaccine effectiveness estimates for community-dwelling adults aged > 65 years, by influenza type/subtype and influenza season (2010/11 to 2015/16)

9. Figure S1. Forest plots of the estimates of current season vaccine effectiveness against influenza A(H3N2) for community-dwelling adults aged > 65 years, taking into account vaccination histories for one, five, and 10 previous seasons by stratifying by (i.e., conditioning on) vaccination history (**panel a**), and correcting for misclassification of current season vaccination status (**panel b**)

10. Figure S2. Forest plots of the estimates of current season vaccine effectiveness against influenza A(H1N1)pdm09 for community-dwelling adults aged > 65 years, taking into account vaccination histories for one, five, and 10 previous seasons by stratifying by (i.e., conditioning on) vaccination history (**panel a**), and correcting for misclassification of current season vaccination status (**panel b**)

11. Figure S3. Forest plots of the estimates of current season vaccine effectiveness against influenza B for community-dwelling adults aged > 65 years, taking into account vaccination histories for one, five, and 10 previous seasons by stratifying by (i.e., conditioning on) vaccination history (**panel a**), and correcting for misclassification of current season vaccination status (**panel b**)

12. Figure S4. Forest plots of the estimates of current season vaccine effectiveness against any influenza restricted to community-dwelling adults aged ≥ 75 years taking into account vaccination histories for one, five, and 10 previous seasons, by stratifying by (i.e., conditioning on) vaccination history (**panel a**), and correcting for misclassification of current season vaccination status (**panel b**)

13. Figure S5. Forest plots of the estimates of current season vaccine effectiveness against any influenza for community-dwelling adults aged > 65 years, taking into account vaccination histories for one, five, and 10 previous seasons by stratifying by (i.e., conditioning on) vaccination history and, based on misclassification of current season vaccination status, manually reclassifying vaccination status from unvaccinated to vaccinated for all previous seasons (**panel a**), and moving individuals 'up' a single category (**panel b**)

14. Figure S6. Forest plots of the estimates of current season vaccine effectiveness against any influenza for community-dwelling adults aged > 65 years, taking into account vaccination histories for one, five, and 10 previous seasons, using the standard approach of a common reference group (i.e., patients who were not vaccinated in the current or any previous seasons under consideration) (**panel a**), and correcting for misclassification of current season vaccination status (**panel b**)

Table S1. Participating laboratories and research ethics approval numbers

Laboratory	Ethics approval numbers
Children's Hospital of Eastern Ontario	14/124X
London Health Sciences Centre	106495
Mount Sinai Hospital	14-0142-C
North York General Hospital	14-0028
Public Health Ontario	2013-051.04
St. Joseph's Healthcare Hamilton	14-785-C
Sunnybrook Health Sciences Centre	082-2014
University Health Network	14-7887.6
William Osler Health System	14-0014

Table S2. Descriptive characteristics of individuals with subtyped and unsubtyped influenza A specimens obtained from community-dwelling adults aged > 65 years, 2010/11 to 2015/16 influenza seasons (n=9,364)

	N (%)		
**Characteristic**	**Subtyped Specimens (n=4,592)**	**Not Subtyped Specimens (n=4,772)**	**p-value**
Influenza season			< 0.001
2010/11	807 (17.6)	331 (6.9)	
2011/12	80 (1.7)	28 (0.6)	
2012/13	999 (21.8)	1,130 (23.7)	
2013/14	371 (8.1)	350 (7.3)	
2014/15	1,787 (38.9)	2,290 (48.0)	
2015/16	548 (11.9)	643 (13.5)	
Age (years), mean ± SD	80.2 ± 8.1	80.5 ± 8.1	0.076
Age group in years			0.24
66-75	1,395 (30.4)	1,411 (29.6)	
76-85	1,886 (41.1)	1,923 (40.3)	
≥ 86	1,311 (28.5)	1,438 (30.1)	
Male sex	2,171 (47.3)	2,218 (46.5)	0.44
Neighbourhood income quintile			0.012
1 (lowest)	1,086 (23.6)	1,087 (22.8)	
2	1,025 (22.3)	944 (19.8)	
3	847 (18.4)	944 (19.8)	
4	807 (17.6)	917 (19.2)	
5 (highest)	804 (17.5)	849 (17.8)	
Missing	23 (0.5)	31 (0.6)	
Received homecare services, past 1y	2,159 (47.0)	2,143 (44.9)	0.041
Hospitalisations, past 3y, mean ± SD	1.3 ± 2.0	1.3 ± 2.0	0.49
Outpatient visits, past 1y, mean ± SD	13.9 ± 11.1	12.2 ± 9.6	< 0.001
Prescription medications, past 1y, mean ± SD	15.8 ± 9.3	15.5 ± 8.9	0.049
Month of influenza testing			< 0.001
November	84 (1.8)	40 (0.8)	
December	1,581 (34.4)	1,149 (24.1)	
January	1,646 (35.8)	1,967 (41.2)	
February	685 (14.9)	889 (18.6)	
March	430 (9.4)	615 (12.9)	
April	130 (2.8)	96 (2.0)	
May	36 (0.8)	16 (0.3)	
Vaccinated against influenza	2,127 (46.3)	2,425 (50.8)	< 0.001

Supplementary Text. Sensitivity analysis involving manual reclassification of past vaccination status based on misclassification of current season vaccination status

For each execution of the misclassification macro developed by Fox et al.,(1) thousands of iterations of exposure re-classification are performed on the observed data, with each iteration using a different combination of sensitivity and specificity values within the pre-specified ranges to calculate an OR. Thus, some individuals could be deemed misclassified in one iteration but not the next. The reported misclassification-corrected adjusted vaccine effectiveness (VE) estimate is based on the median odds ratio (OR) calculated from all iterations.

We modified the macro to retain the re-classified current season vaccination status for each individual from each iteration, which we used to determine the overall proportion vaccinated separately among test-positive cases and test-negative controls for each iteration. After all iterations were completed, we calculated the proportion of iterations for which each individual was re-classified as vaccinated. We also calculated the median overall proportion vaccinated for cases and controls. If an individual's proportion was greater than the median for their case status, their current season vaccination status was set to vaccinated.

For those individuals whose current season vaccination status was re-classified, we created two scenarios to also change their past seasons' vaccination history. In the first scenario, we assumed that for all previous seasons where they were considered to be unvaccinated in the administrative data, we changed them to vaccinated. Consequently, these individuals were re-categorized to the highest past vaccination history group in each respective analysis (e.g., in the one previous season analysis, all were re-categorized into the 'vaccinated in the previous season' group; in the five previous seasons analysis, all were re-categorized to the 'vaccinated in 4-5 previous seasons' group). In the second scenario, we assumed that only some of the previous seasons were misclassified as unvaccinated. To achieve this, we moved these individuals 'up' one past vaccination history category (e.g., for the analysis examining 5-year vaccination history, those initially considered vaccinated in none of the previous five seasons were re-categorized to the 'vaccinated in 1-3 of the previous five seasons' group). We conducted stratified analyses using these new past vaccination history groups for both scenarios.

Reference

Fox MP, Lash TL, Greenland S. A method to automate probabilistic sensitivity analyses of misclassified binary variables. Int J Epidemiol. 2005 Dec 1;34(6):1370–6.

Table S3. Descriptive characteristics of influenza test-positive and influenza test-negative community-dwelling adults aged > 65 years, 2010/11 to 2015/16 influenza seasons (n=58,304)

Characteristic	N (%) Test-Positive (n=11,496)	N (%) Test-Negative (n=46,808)	p-value
Influenza season*			< 0.001
2010/11	1,204 (10.5)	4,980 (10.6)	
2011/12	413 (3.6)	3,216 (6.9)	
2012/13	2,253 (19.6)	8,577 (18.3)	
2013/14	1,554 (13.5)	9,665 (20.6)	
2014/15	4,432 (38.6)	12,044 (25.7)	
2015/16	1,640 (14.3)	8,326 (17.8)	
Age* (years), mean ± SD	80.2 ± 8.2	79.4 ± 8.1	< 0.001
Age group in years			< 0.001
66-75	3,601 (31.3)	16,716 (35.7)	
76-85	4,548 (39.6)	18,181 (38.8)	
≥86	3,347 (29.1)	11,911 (25.4)	
Male sex*	5,348 (46.5)	22,446 (48.0)	0.006
Neighbourhood income quintile*			0.036
1 (lowest)	2,630 (22.9)	10,473 (22.4)	
2	2,475 (21.5)	9,909 (21.2)	
3	2,177 (18.9)	8,805 (18.8)	
4	2,088 (18.2)	8,308 (17.7)	
5 (highest)	2,062 (17.9)	9,022 (19.3)	
Missing	64 (0.6)	291 (0.6)	
Medical conditions			
Cardiovascular disease[a]	7,021 (61.1)	30,293 (64.7)	< 0.001
Chronic obstructive pulmonary disease	5,376 (46.8)	24,378 (52.1)	< 0.001
Diabetes	5,020 (43.7)	19,930 (42.6)	0.034
Cancer	2,877 (25.0)	14,244 (30.4)	< 0.001
Asthma	3,102 (27.0)	13,112 (28.0)	0.027
Anaemia	2,445 (21.3)	11,581 (24.7)	< 0.001
Chronic kidney disease	2,331 (20.3)	10,546 (22.5)	< 0.001
Dementia/frailty	2,505 (21.8)	8,947 (19.1)	< 0.001
Immunocompromised	1,363 (11.9)	6,843 (14.6)	< 0.001
Any of the above medical conditions*	10,806 (94.0)	44,773 (95.7)	< 0.001
Received homecare services*, past 1y	5,193 (45.2)	23,203 (49.6)	< 0.001
Hospitalisations*, past 3y, mean ± SD	1.3 ± 1.9	1.7 ± 2.3	< 0.001
Outpatient visits*, past 1y, mean ± SD	13.0 ± 10.4	14.5 ± 11.1	< 0.001
Prescription medications*, past 1y, mean ± SD	15.5 ± 9.0	16.8 ± 9.4	< 0.001
Month of influenza testing*			< 0.001
November	127 (1.1)	1,284 (2.7)	
December	2,763 (24.0)	6,763 (14.4)	
January	3,676 (32.0)	11,420 (24.4)	
February	1,780 (15.5)	8,565 (18.3)	
March	1,802 (15.7)	8,953 (19.1)	
April	1,057 (9.2)	6,593 (14.1)	
May	291 (2.5)	3,230 (6.9)	
Tested sample from inpatient setting	9,170 (79.8)	40,695 (86.9)	< 0.001
Vaccinated against influenza	5,575 (48.5)	25,429 (54.3)	< 0.001
Vaccinated in prior season	6,177 (53.7)	27,068 (57.8)	< 0.001

* Included in multivariable models for estimating vaccine effectiveness

[a] Includes acute ischaemic stroke, arrhythmias, congestive heart failure, ischaemic heart disease, and transient ischaemic attack.

Table S4. Descriptive characteristics of vaccinated and unvaccinated community-dwelling adults aged > 65 years, 2010/11 to 2015/16 influenza seasons (n=58,304)

Characteristic	N (%) Vaccinated (n=31,004)	N (%) Unvaccinated (n=27,300)	p-value
Influenza season*			< 0.001
2010/11	3,049 (9.8)	3,135 (11.5)	
2011/12	2,018 (6.5)	1,611 (5.9)	
2012/13	5,327 (17.2)	5,503 (20.2)	
2013/14	6,079 (19.6)	5,140 (18.8)	
2014/15	9,128 (29.4)	7,348 (26.9)	
2015/16	5,403 (17.4)	4,563 (16.7)	
Age* (years), mean ± SD	80.0 ± 7.9	79.0 ± 8.4	< 0.001
Age group in years			< 0.001
66-75	9,912 (32.0)	10,405 (38.1)	
76-85	12,730 (41.1)	9,999 (36.6)	
≥86	8,362 (27.0)	6,896 (25.3)	
Male sex*	15,081 (48.6)	12,713 (46.6)	< 0.001
Neighbourhood income quintile*			< 0.001
1 (lowest)	6,665 (21.5)	6,438 (23.6)	
2	6,589 (21.3)	5,795 (21.2)	
3	5,860 (18.9)	5,122 (18.8)	
4	5,586 (18.0)	4,810 (17.6)	
5 (highest)	6,147 (19.8)	4,937 (18.1)	
Missing	157 (0.5)	198 (0.7)	
Medical conditions			
Cardiovascular disease[a]	20,258 (65.3)	17,056 (62.5)	< 0.001
Chronic obstructive pulmonary disease	16,561 (53.4)	13,193 (48.3)	< 0.001
Diabetes	13,595 (43.8)	11,355 (41.6)	< 0.001
Cancer	9,290 (30.0)	7,831 (28.7)	< 0.001
Asthma	9,340 (30.1)	6,874 (25.2)	< 0.001
Anaemia	7,673 (24.7)	6,353 (23.3)	< 0.001
Chronic kidney disease	6,874 (22.2)	6,003 (22.0)	0.578
Dementia/frailty	5,829 (18.8)	5,623 (20.6)	< 0.001
Immunocompromised	4,599 (14.8)	3,607 (13.2)	< 0.001
Any of the above medical conditions*	29,808 (96.1)	25,771 (94.4)	< 0.001
Received homecare services*, past 1y	14,523 (46.8)	13,873 (50.8)	< 0.001
Hospitalisations*, past 3y, mean ± SD	1.5 ± 2.1	1.7 ± 2.3	< 0.001
Outpatient visits*, past 1y, mean ± SD	15.6 ± 10.8	12.6 ± 10.9	< 0.001
Prescription medications*, past 1y, mean ± SD	17.2 ± 8.9	15.7 ± 9.6	< 0.001
Month of influenza testing*			< 0.001
November	509 (1.6)	902 (3.3)	
December	4,674 (15.1)	4,852 (17.8)	
January	7,919 (25.5)	7,177 (26.3)	
February	5,592 (18.0)	4,753 (17.4)	
March	5,969 (19.3)	4,786 (17.5)	
April	4,329 (14.0)	3,321 (12.2)	
May	2,012 (6.5)	1,509 (5.5)	
Tested sample from inpatient setting	26,583 (85.7)	23,282 (85.3)	0.116
Specimen positive for influenza	5,575 (18.0)	5,921 (21.7)	< 0.001
Vaccinated in prior season	24,594 (79.3)	8,651 (31.7)	< 0.001

* Included in multivariable models for estimating vaccine effectiveness
[a] Includes acute ischaemic stroke, arrhythmias, congestive heart failure, ischaemic heart disease, and transient ischaemic attack.

Table S5. Influenza vaccine effectiveness estimates for community-dwelling adults aged > 65 years, by influenza type/subtype and influenza season (2010/11 to 2015/16)[a]

Analysis	Test-Positive, No. Vaccinated/Total	Test-Negative, No. Vaccinated/Total	Unadjusted VE% (95% CI)	Adjusted VE% (95% CI)
2010/11				
Influenza A				
A(H1N1)pdm09	≤ 5[b] / ≤ 26[b]	2,561 / 4,980	76 (37, 91)	72 (25, 90)
A(H3N2)	311 / 782	2,561 / 4,980	38 (27, 47)	33 (21, 43)
A(unsubtyped)	144 / 331	2,561 / 4,980	27 (9, 42)	26 (7, 42)
Influenza B	28 / 66	2,561 / 4,980	30 (–14, 57)	35 (–8, 61)
2011/12				
Influenza A				
A(H1N1)pdm09	14 / 33	1,823 / 3,216	44 (–13, 72)	31 (–41, 66)
A(H3N2)	22 / 47	1,823 / 3,216	33 (–20, 62)	40 (–9, 67)
A(unsubtyped)	18 / 28	1,823 / 3,216	–38 (–199, 37)	–47 (–229, 35)
Influenza B	141 / 305	1,823 / 3,216	34 (17, 48)	35 (17, 50)
2012/13				
Influenza A				
A(H1N1)pdm09	29 / 47	4,339 / 8,577	–57 (–184, 13)	–59 (–191, 14)
A(H3N2)	421 / 953	4,339 / 8,577	23 (12, 32)	20 (7, 30)
A(unsubtyped)	484 / 1,130	4,339 / 8,577	27 (17, 35)	23 (12, 33)
Influenza B	54 / 124	4,339 / 8,577	25 (–8, 47)	28 (–4, 50)
2013/14				
Influenza A				
A(H1N1)pdm09	103 / 269	5,368 / 9,665	50 (36, 61)	44 (28, 57)
A(H3N2)	54 / 103	5,368 / 9,665	12 (-30, 40)	21 (–17, 47)
A(unsubtyped)	160 / 350	5,368 / 9,665	33 (17, 46)	26 (7, 40)
Influenza B	394 / 834	5,368 / 9,665	28 (17, 38)	38 (28, 47)
2014/15				
Influenza A				
A(H1N1)pdm09	≤ 5[b] / ≤ 5[b]	6,712 / 12,044	47 (–217, 91)	31 (–346, 89)
A(H3N2)	926 / 1,783	6,712 / 12,044	14 (5, 22)	17 (7, 25)
A(unsubtyped)	1,302 / 2,290	6,712 / 12,044	–5 (–15, 4)	–5 (–16, 4)
Influenza B	186 / 356	6,712 / 12,044	13 (–7, 30)	18 (–3, 34)
2015/16				
Influenza A				
A(H1N1)pdm09	194 / 451	4,626 / 8,326	40 (27, 50)	37 (24, 49)
A(H3N2)	46 / 97	4,626 / 8,326	28 (–8, 52)	31 (–3, 54)
A(unsubtyped)	317 / 643	4,626 / 8,326	22 (9, 34)	17 (2, 30)
Influenza B	224 / 453	4,626 / 8,326	22 (5, 35)	22 (6, 36)

[a] The model adjusted for age, sex, census area-level neighbourhood income quintile, number of hospitalisations in the past 3 years, number of outpatient visits in the past year, receipt of home care services in the past year, number of prescription medications in the past year, comorbidities that increase the risk of influenza complications (anaemia, cancer, cardiovascular disease, dementia, diabetes, frailty, immunodeficiency due to underlying disease and/or therapy, as well as renal disease and respiratory disease), and calendar time.
[b] Suppressed due to small cell sizes (direct or by inference), which cannot be reported as per privacy regulations.

Figure S1. Forest plots of (A) current season vaccine effectiveness estimates against influenza A(H3N2) for community-dwelling adults aged > 65 years, taking into account vaccination histories for one, five, and 10 previous seasons and stratifying according to number of vaccinations received, and (B) also correcting for misclassification of current season vaccination status

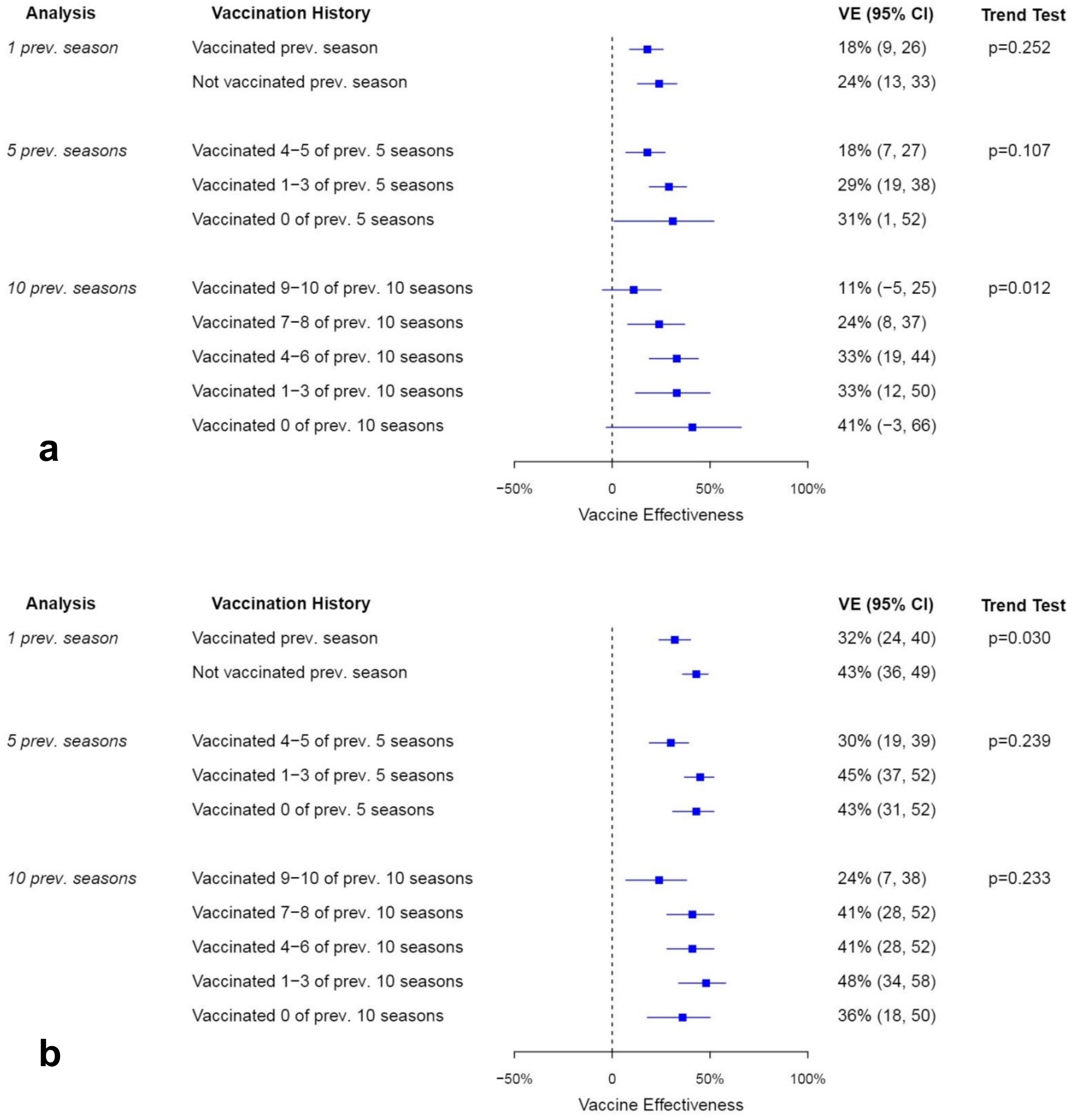

[a] The model adjusted for age, sex, census area-level neighbourhood income quintile, number of hospitalisations in the past 3 years, number of outpatient visits in the past year, receipt of home care services in the past year, number of prescription medications in the past year, comorbidities that increase the risk of influenza complications (anaemia, cancer, cardiovascular disease, dementia, diabetes, frailty, immunodeficiency due to underlying disease and/or therapy, as well as renal disease and respiratory disease), calendar time, and influenza season.

Figure S2. Forest plots of (A) current season vaccine effectiveness estimates against influenza A(H1N1)pdm09 for community-dwelling adults aged > 65 years, taking into account vaccination histories for one, five, and 10 previous seasons and stratifying according to number of vaccinations received, and (B) also correcting for misclassification of current season vaccination status

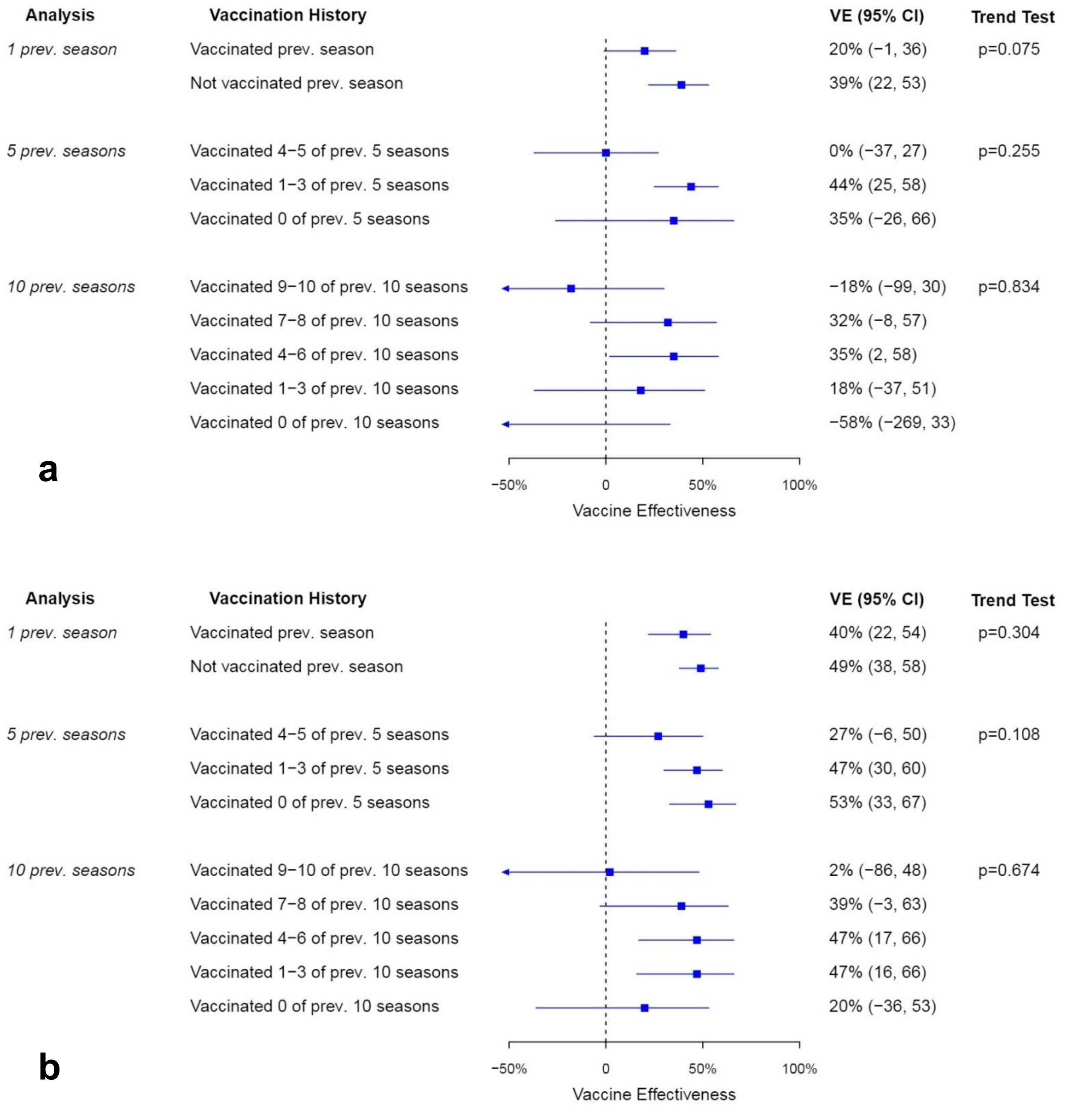

[a] The model adjusted for age, sex, census area-level neighbourhood income quintile, number of hospitalisations in the past 3 years, number of outpatient visits in the past year, receipt of home care services in the past year, number of prescription medications in the past year, comorbidities that increase the risk of influenza complications (anaemia, cancer, cardiovascular disease, dementia, diabetes, frailty, immunodeficiency due to underlying disease and/or therapy, as well as renal disease and respiratory disease), calendar time, and influenza season.

Figure S3. Forest plots of (A) current season vaccine effectiveness estimates against influenza B for community-dwelling adults aged > 65 years, taking into account vaccination histories for one, five, and 10 previous seasons and stratifying according to number of vaccinations received, and (B) also correcting for misclassification of current season vaccination status

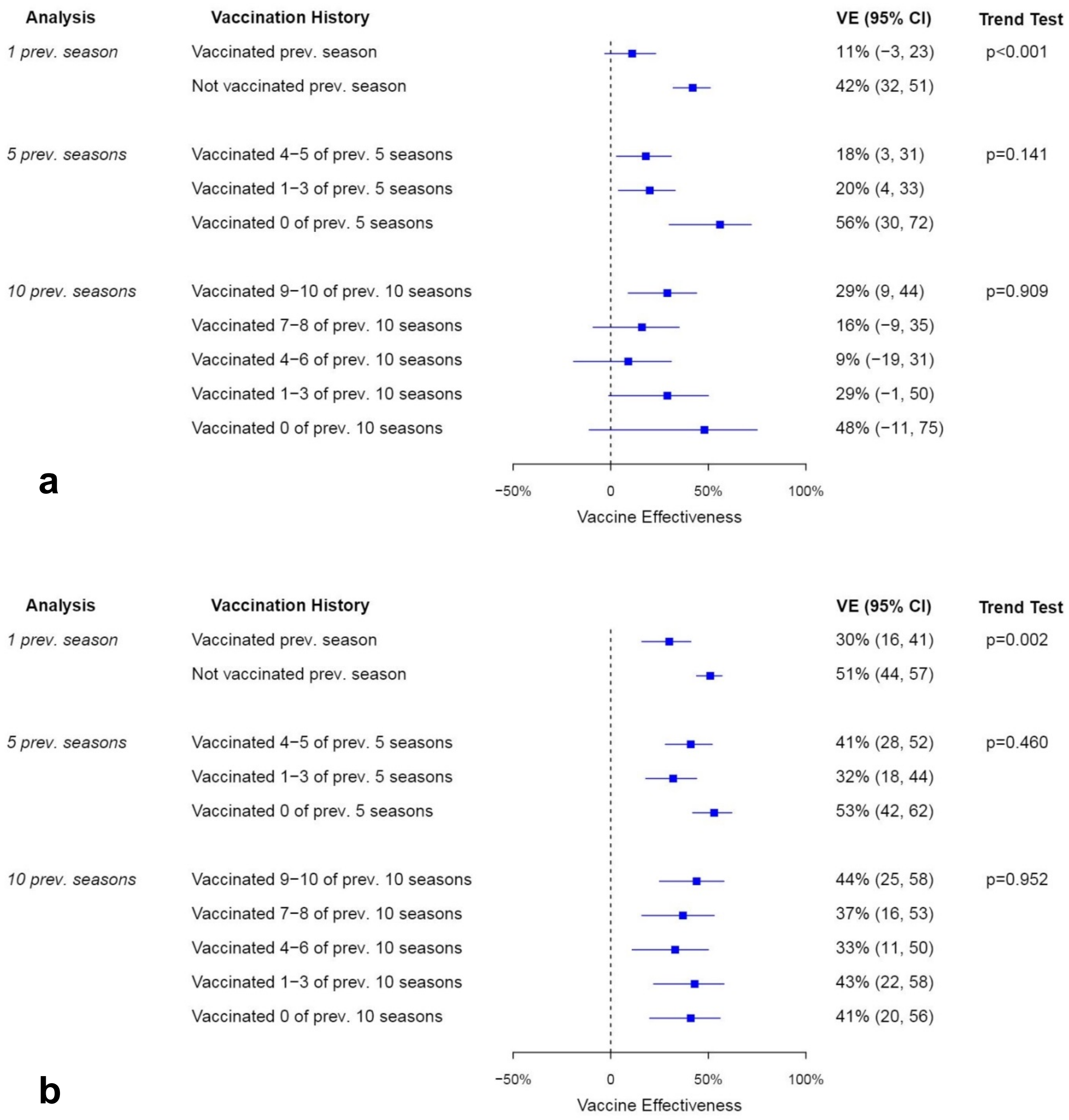

[a] The model adjusted for age, sex, census area-level neighbourhood income quintile, number of hospitalisations in the past 3 years, number of outpatient visits in the past year, receipt of home care services in the past year, number of prescription medications in the past year, comorbidities that increase the risk of influenza complications (anaemia, cancer, cardiovascular disease, dementia, diabetes, frailty, immunodeficiency due to underlying disease and/or therapy, as well as renal disease and respiratory disease), calendar time, and influenza season.

Figure S4. Forest plots of (A) current season vaccine effectiveness estimates against any influenza restricted to community-dwelling adults aged ≥ 75 years, taking into account vaccination histories for one, five, and 10 previous seasons and stratifying according to number of vaccinations received, and (B) also correcting for misclassification of current season vaccination status

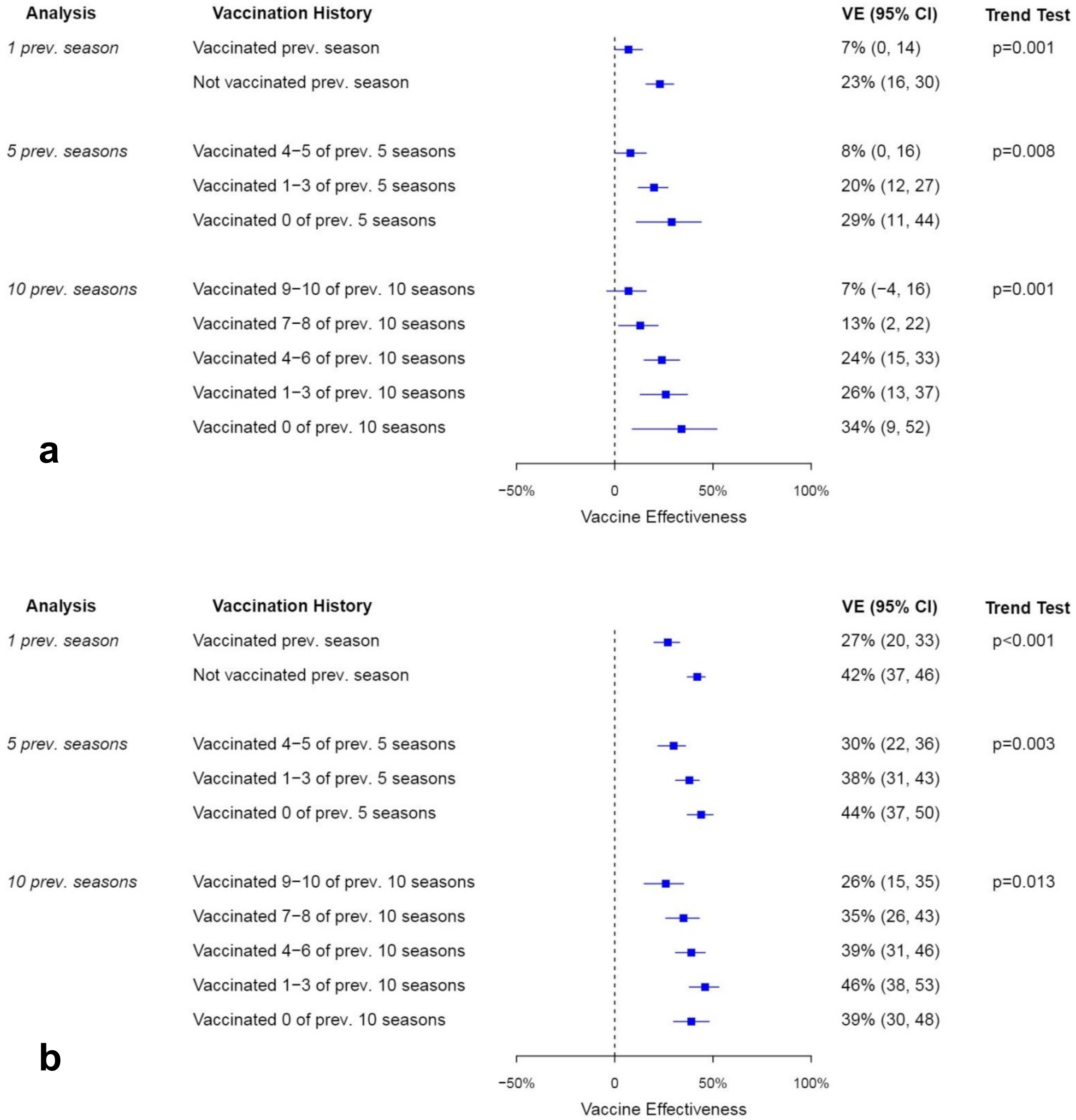

[a] The model adjusted for age, sex, census area-level neighbourhood income quintile, number of hospitalisations in the past 3 years, number of outpatient visits in the past year, receipt of home care services in the past year, number of prescription medications in the past year, comorbidities that increase the risk of influenza complications (anaemia, cancer, cardiovascular disease, dementia, diabetes, frailty, immunodeficiency due to underlying disease and/or therapy, as well as renal disease and respiratory disease), calendar time, and influenza season.

Figure S5. Forest plots of current season vaccine effectiveness estimates against any influenza for community-dwelling adults aged > 65 years, taking into account vaccination histories for one, five, and 10 previous seasons and stratifying according to number of vaccinations received and, based on misclassification of current season vaccination status, (A) manually reclassifying vaccination status from unvaccinated to vaccinated for all previous seasons, and (B) moving individuals ‘up’ a single category

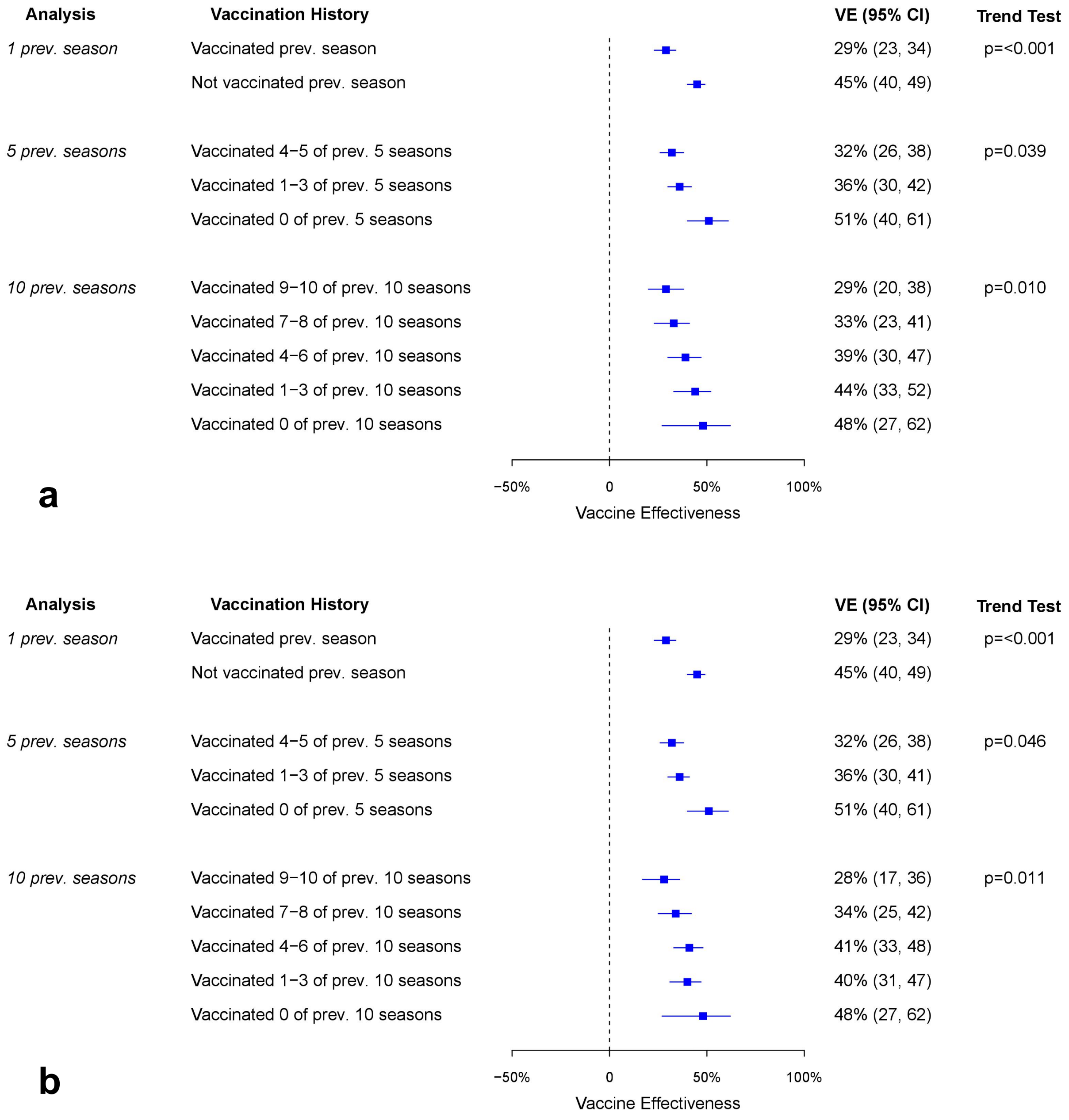

[a] The model adjusted for age, sex, census area-level neighbourhood income quintile, number of hospitalisations in the past 3 years, number of outpatient visits in the past year, receipt of home care services in the past year, number of prescription medications in the past year, comorbidities that increase the risk of influenza complications (anaemia, cancer, cardiovascular disease, dementia, diabetes, frailty, immunodeficiency due to underlying disease and/or therapy, as well as renal disease and respiratory disease), calendar time, and influenza season.

Figure S6. Forest plots of (A) current season vaccine effectiveness estimates against any influenza for community-dwelling adults aged > 65 years, taking into account vaccination histories for one, five, and 10 previous seasons, using the standard approach of a common reference group (i.e., patients who were not vaccinated in the current or any previous seasons under consideration), and (B) also correcting for misclassification of current season vaccination status

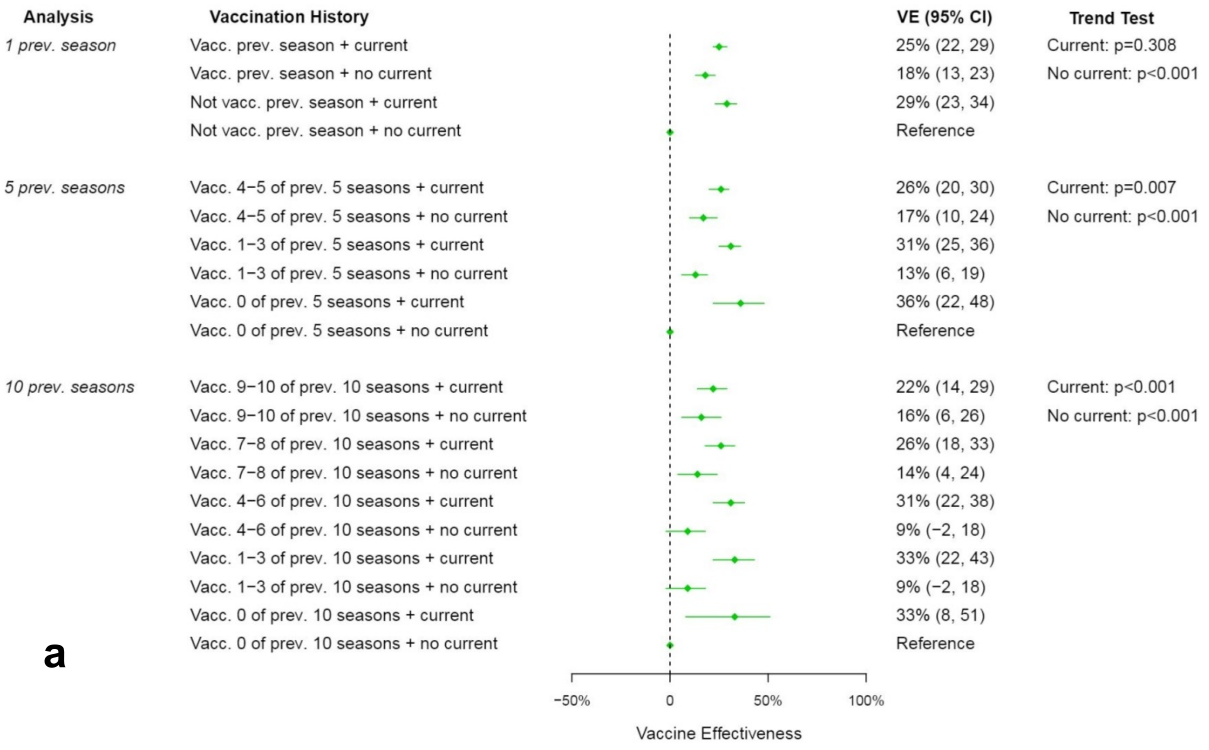

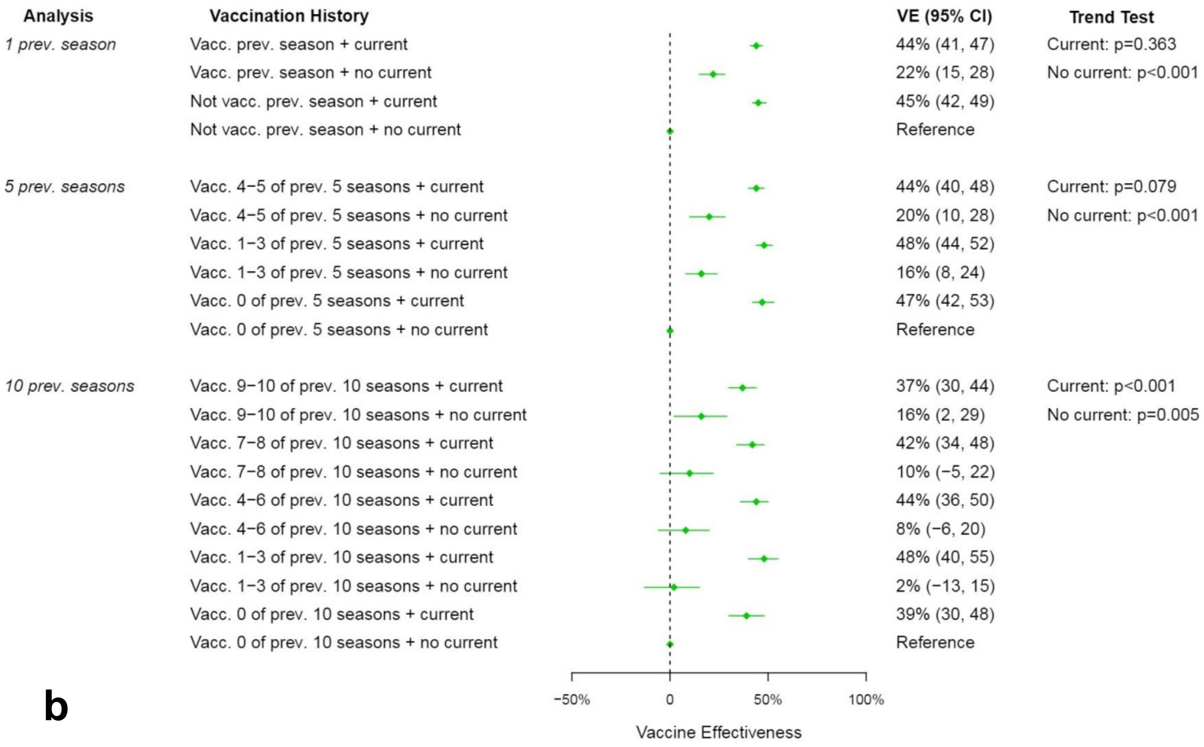

b

[a] The model adjusted for age, sex, census area-level neighbourhood income quintile, number of hospitalisations in the past 3 years, number of outpatient visits in the past year, receipt of home care services in the past year, number of prescription medications in the past year, comorbidities that increase the risk of influenza complications (anaemia, cancer, cardiovascular disease, dementia, diabetes, frailty, immunodeficiency due to underlying disease and/or therapy, as well as renal disease and respiratory disease), calendar time, and influenza season.
